# Supporting health and wellbeing in health care employees: a documentary review of organizational policies, strategies and frameworks

**DOI:** 10.3389/fspor.2024.1308603

**Published:** 2024-05-30

**Authors:** Louise Patricia Hoyle, Gemma Cathrine Ryde, Jamie Coulter, Jennie Rollason

**Affiliations:** ^1^ Faculty of Health Science and Sport, University of Stirling, Stirling, United Kingdom; ^2^ School of Cardiovascular and Metabolic Health, University of Glasgow, Glasgow, United Kingdom; ^3^ Macmillan Cancer Support, London, United Kingdom; ^4^ Cambridge University Hospitals NHS Foundation Trust, Cambridge, United Kingdom

**Keywords:** health care, physical activity, policy, documentary analysis, health care staff

## Abstract

**Background:**

Health care workers are crucial for a productive and thriving health care system, yet the health and lifestyle behaviour of key groups within this workforce (for example nurses and healthcare assistants/support workers) is typically poor. The extent of health and wellbeing documents that guide action towards improving their health and wellbeing is unknown. Using one health care system, NHS Scotland, as an example, the aim of this study was to assess the number of NHS health boards with workplace documents focused on health and wellbeing of employees, the quality of these documents and the extent to which they reference lifestyle behaviours, namely physical activity.

**Methods:**

Documentary analysis was undertaken on employee health and wellbeing policies (and wider documents). These were sourced through online searches on Google search engine and Freedom of Information Requests sent to all 14 Scottish NHS Health Boards. Titles and content were assessed for relevance to employee health and wellbeing. Content analysis was used to analyse the included documents against eight predefined codes.

**Results:**

Thirteen documents were retrieved with 11 of the 14 Health Boards having at least one relevant document. The content varied greatly between documents with regards to how many reported the eight codes and the quality of content within these. Nine documents mentioned physical activity but mainly in relation to current activities rather than in the context of a future healthy workforce.

**Conclusions:**

Despite the importance of a healthy, health care workforce, more work is needed to ensure high level documents are able to support these efforts, especially with reference to lifestyle behaviours.

## Introduction

Health care employees are essential to a productive and thriving health care system worldwide. The health care workforce is vast, with an estimated 65 million employees globally (1). This workforce is essential for delivering high-quality patient care and help deliver key global agendas such as the United Nations (UN) Sustainable Development Goals with The World Health Organisation (WHO) Working for Health 2022–2030 action plan emphasizing their importance for population health, health system sustainability, economic prosperity and social protection (2). Despite this, the health of this workforce is typically poor (3) with health care employees more likely to have increased body mass indices, are at higher risk of developing obesity, type 2 diabetes, and coronary artery disease than the general population (4–7). The world's largest employers of health care staff, the National Health Service (NHS), with 1.7 million employees across the United Kingdom (8–11), report challenges within the workforce as a result of employee health including higher levels of sickness absence and work-related illness than those in other sectors. For example, in 2021, NHS England reported a sickness absence rate of ranging between 4.1% and 6.2% (12) compared to the 2.2% national rate, with other home nations such as Scotland reporting similar figures (13). In many European Countries, sickness absence is also high among healthcare employees (14). Focusing on the health of this workforce is therefore a key priority area.

Whilst multiple factors contribute towards employee health and wellbeing, tackling lifestyle behaviours that affect both health care employees and the wider population is essential (15). Lifestyle behaviours such as smoking, alcohol use, diet and physical inactivity are significant contributors to noncommunicable diseases, health and wellbeing (16) and can impact on many of the health conditions that disproportionality effect health care employees. Health care employees are also key messengers, advocates and promoters of lifestyle behaviors/health at a population, with health care employees who practice healthy lifestyle behaviours more likely to promote them with patients (17, 18). A significant number of interventions to improve health care employee health have been implemented as a result for example in 2020 a rapid review and evidence map of workplace-based interventions to promote healthy lifestyles in healthcare employees was undertaken and yielded 12 review of reviews and 312 other reviews (19). Interventions varied widely and included exercise programs, nutritional advice, lifestyle education sessions and e-health messaging (20), but authors concluded that research was typically of poor quality and that there was no best practice intervention approach recommend for this setting. All included interventions were also mainly individually focused and neglected the wider contents that can support interventions in this setting. Numerous potential interventions are therefore available that could improve NHS employee health and wellbeing, however, the best way to implement these is still unknown.

One area that is largely under researched with regards to the health and wellbeing of health care employees is the organizational policy environment. With a move towards more ecological and systems-based approaches which reflects the wider cultural, environmental and organizational policy aspects around employee health and wellbeing (21, 22) there is a need to explore the role of organizational policy within this. This is different to public policy such as smoking bans or seatbelt usage which is established by governments and are typically related to legislation (23). To date, the extent to which organizational health and wellbeing policies endorse and guide action towards improved health care employee health and wellbeing is unknown. In addition, whether these documents provide support for promoting healthy lifestyle behaviours known to benefit positive health outcomes is also unclear. Whilst it might not always be the case that organizational policy equates to action or improvement in employee health, there is evidence that organizational policy can positively influence health behaviours (24, 25).

This study uses one health care system, NHS Scotland (26), as an example to explore organizational health and wellbeing policies for health care employees and the extent to which they focus on promoting lifestyle behaviours. Whilst there are numerous lifestyle behaviours that could be explored, this paper will focus on physical activity. Physical activity is defined as any bodily movement produced by skeletal muscles that results in energy expenditure (27). Physical activity therefore incorporates a wide range of activities such as walking, cycling, gardening and lifting weights and these are normally described within the domain in which they occur—transport, domestic/household, leisure time, occupational. Physical activity is especially important in health care workers and has been shown to improve stress, burnout and wellbeing (28, 29), depression and anxiety (30) blood pressure, vitality and life satisfaction (31). Therefore, out of all the possible health behaviours, physical activity has a significant potential to influence a wide range of health and other outcomes (physical, mental, social and organizational) which are essential in this workplace. Whilst, some health care workers can gain a large amount of movement (mainly steps) at work, evidence suggest that occupational activity may not confer the same benefits as other domains, namely leisure time physical activity (32) and therefore is still an important area to explore in this workforce.

The aim of this study is therefore to assess; (1) the number of NHS Scotland health boards that have organizational policies focused on health and wellbeing of employees and the quality of these documents; and (2) the extent to which these reference lifestyle behaviours, namely physical activity.

## Methods

This study reviewed organizational policies (and wider documents) written by NHS managers/Human Resources with the function of supporting the health and wellbeing of NHS employees. The definition of health policy was based on the World Health Organization (WHO) guidance (33):

*Health policy refers to decisions, plans, and actions that are undertaken to achieve specific health care goals within a society. An explicit health policy can achieve several things: it defines a vision for the future which in turn helps to establish targets and points of reference for the short and medium term. It outlines priorities and the expected roles of different groups; and it builds consensus and informs people*.

These documents are not available through peer-reviewed literature and are typically published on NHS websites, employee intranets or portals. Therefore, a conventional review and analysis was not applicable for this study. Instead, Documentary Analysis (or document review) was used and is a common approach in health policy research (34). The READ approach provides a framework for document analysis and was applied in this study (35). This approach consisted of four steps: (1) readying the materials, (2) extracting the data, (3) analysis/synthesizing the and (4) distilling the findings. Ethical approval for this study was granted by General University Ethics Panel, University of Stirling; Reference Number EC 20224791 5015.

### Information sources and search strategy

An initial search was conducted using the Google search engine in July 2019 to assess whether documents were available online without the need to contact NHS boards directly. Search terms included the name of the 14 territorial NHS Scotland Health Boards and terms related to the population (employees), intervention (health and wellbeing) and document type (policy). No date range were applied to the search. From this initial search it was clear that some documents were visible online but not accessible such as those on staff intranet sites and that some health and wellbeing documents were incorporated into other policies and strategies and might not be discovered in an online search alone or by only focusing on documents classed as “policy”. A broader definition of a “policy” was therefore adopted to include “*formal written codes, strategies, plans, decisions, regulations and directives that have been official enacted or endorsed by the governing body at a given level”* (36). Therefore, the search was broadened to include terms such as “strategy” and “framework”.

A secondary search approach was adopted to gain access to documents that were visible online but not accessible and to check for other documents that might not be posted online. A Freedom of Information request (FOI) was sent to all 14 territorial NHS Scotland health boards in July 2019, requesting and policy/strategy/framework (other document) within the named Health Board which focus on employee health and wellbeing. Due to the time between the initial FOI and data analysis a further FOI was sent in November 2021 and a follow-up one in March 2022 to ensure the most recent documents were captured.

### Eligibility criteria and screening

Document titles were entered into a spreadsheet in Microsoft Excel. Duplicates were removed and titles screened for relevance by Author 2 (10% were then checked by Author 4 & Author 1). Documents were eligible if they were either a policy/strategy/framework that had a primary focus on employee health and wellbeing. Documents were excluded if they did not focus on wellbeing, were not specifically for NHS staff, had been superseded with a newer document (some documents were retrieved via online search, but newer versions were received following FOI requests) or were not a policy, strategy, or framework. Full texts of all remaining documents were assessed for eligibility by Author 2 and were cross checked by both Author 1 and Author 4. We have made the decision not to provide references for the health board documents, but these can be provided on request to the Authors.

### Data extraction, synthesis and analysis

A specific data extraction template and codes were developed for this review and created in Microsoft Excel. The template and codes draws on the WHO definition of health policy previously stated and existing policy analysis tools and frameworks (33, 37–40) including the Australian State Government Health and Wellbeing Policy example document developed to assist workplaces develop their own health and wellbeing policies- (41).

Codes for analysis were selected based on discussions within the research team which comprised of two NHS nursing registrants (Author 2, Author 3), who were actively employed in the NHS, an academic researcher with a nursing registration (Author 4) and an academic researcher in workplace health and wellbeing (Author 1). The final data extraction template comprised the following eight codes: goals/aims, objectives, responsibilities, communication plan, monitoring outcomes, wider policy context, review date and resources. In Table 1, a list of definitions is provided for each code. In addition to these codes, document type and year of publication were also extracted.

**Table 1:** Data extraction headings and definitions and determination of quality level.

Codes for data extraction	Definition	Quality assessment
Goals/aims	What is to be achieved as a result of the document. They should be clear and specific statements.	Low: No clear or specific statements provides Medium: Stated, but not clear or specific High: Clearly stated, well-articulated and comprehensive
Objectives	Actions the Health Board is going to do to achieve their goal/aim. They should be clear, note how they should be achieved, action orientated and specific statements.	Low: Limited information on the objectives and how they could be achieved Medium: Some information on the objectives and how these could be achieved High: Detailed objectives with a clear timeframe and an overview of how they could be achieved—action-orientated
Responsibilities for action	List of specific people/roles/groups in the organisation and what they need to do to help achieve the document aims/objectives. This would normally be split into staff and managers.	Low: Limited detail provided on who is responsible for what action Medium: Provided an overview of what should be done but vague as to who is responsible for which action High: Clear detail of what should be done how it will be done and by whom
Communication plan	Clear plan of how the actions of the document are communicated to ensure the benefits it seeks to have are known about. For example, this could be *via* staff intranet, leaflets, staff meetings etc.	Low: Limited detail provided Medium: Some details provided but unclear or vague High: Clear and detailed plan highlighting how dissemination of the document and/or actions within the document will occur
Monitoring outcomes	Evaluating the changes that occur if the document is enacted which may include indicators and measures (could also be referred to as surveillance).	Low: Limited indicators and measures identified Medium: Some indicators and measures identified High: Clear and specific indicators and measures identified
Wider policy context	Reference to either other internal or national policies, legislation such as from the health and safety executive or reviews on employee health and wellbeing in the NHS.	Low: Limited detail provided Medium: Referenced local policies only High: Referenced both national and local policies and legislation
Review date	Provides the exact date given when the document will be reviewed.	Low: No clear review date Medium: Review date provided but already overdue for review High: Review date provided and still in date
Resources	Any mention of how the activities or actions within the document will be resourced with a focus on financial support. Reference to previously funded activities were not included.	Low: Limited detail provided on how actions will be resources Medium: Some information on how actions will be resources but no detail on how these resources will be made available High: Clear information on how actions will be resourced with reference to financial support

Each document was read and reviewed by Author 1, Author 4 and Author 2. For each code, a description of the content and extracts from each document were added into the data extraction template. It was noted whether this information was provided, not provided, not expected, or not expected but provided. Due to three different types of documents (policy, framework & strategy) being included in analysis, not all headings were applicable for all documents and hence the “not expected” category was created. For example, strategy documents and frameworks might not have a communication plan included or might not provide a set review date but more a time period that the document covers.

The extracts formed the basis of the quality appraisal. Due to the nature of these documents, conventional quality appraisals were not applicable. Previous health policy research within the food (42) and physical activity sector (39) have assessed the quality of policy documents (defined as the strength and comprehensiveness of the information provided) compared against best practice statement examples. For this review, the strength, comprehensiveness and level of detail of the extracted data was used to compare across all documents by both Author 1 and Author 4 and formed the basis of the quality appraisal. The quality of each code was assessed as low, medium, high or not included, with definitions of these provided in Table 1.

To address the aim focusing on physical activity content, each document was also searched for reference to the promotion of physical activity. Physical activity was added as a code to the data extraction template spreadsheet. Using the PDF/word document search function in each document the following terms were searched; physical activity/activities, exercise/s, sport/s, leisure, walk/ing, active travel/transport, bike, cycling and fitness. Each document was also read in full to check for any other derivatives of these terms. Mention of physical *health* but not physical *activity* were not coded as including physical activity. If physical activity was mentioned but not in reference to its role in the promotion of employee wellbeing, this was also not coded. For example, it was not coded if a document merely stated in a background section that “physical activity is good for health”. Data are presented as a description of content, example extracts, whether walking or active transport were explicitly mentioned (yes/no) and if physical activity was measured or noted as a key outcome (yes/no and details/extract). It was possible to extract data specifically on the domain of active transport, but it was not always possible to identify the domain in which physical activity was referenced. Therefore, not all activity domains are explicitly referred to in the results.

## Results

**Figure 1:**
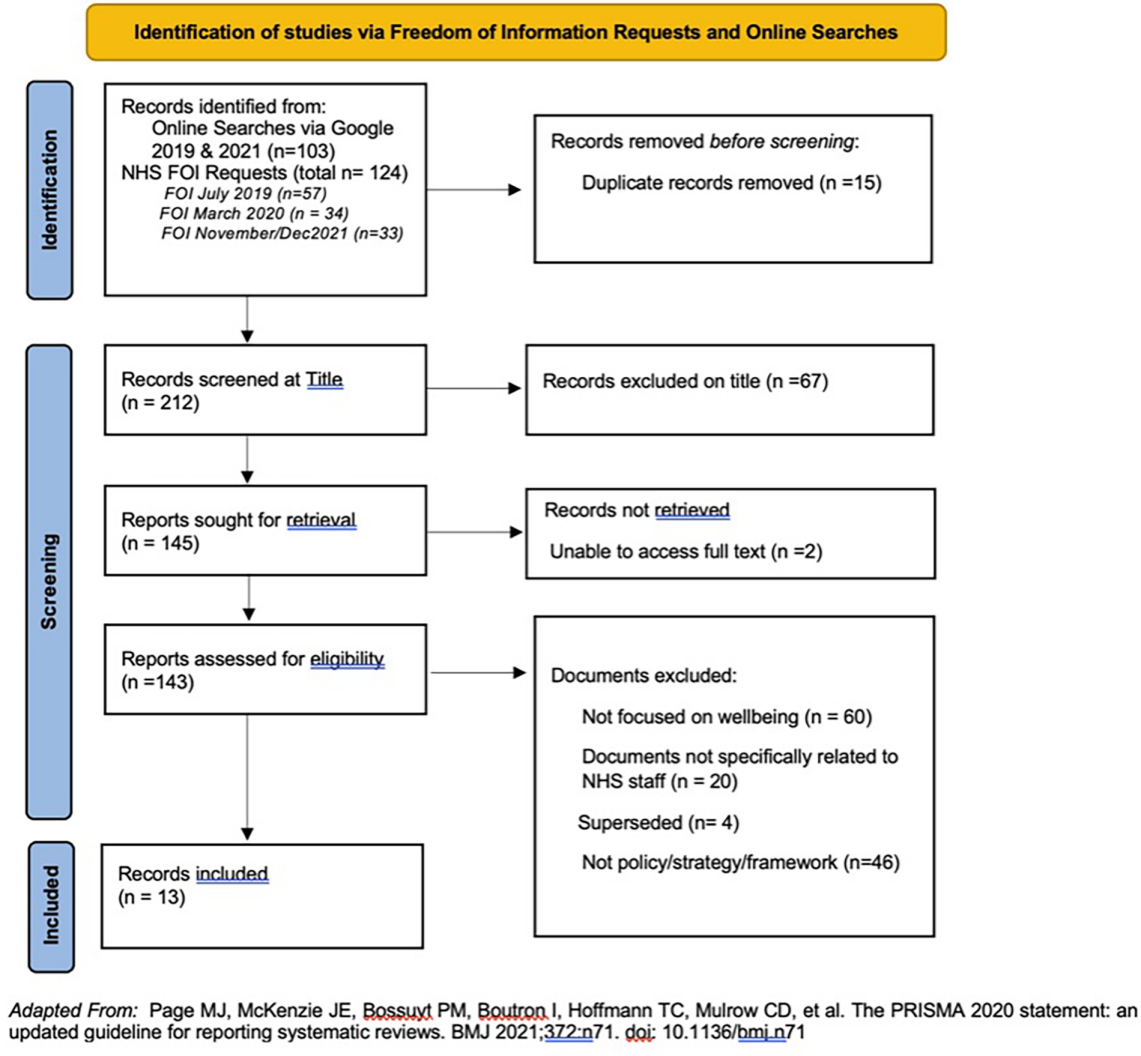
Adapted PRISMA for searches and screening.

Thirteen documents were retrieved (see Figure 1) that focused on employee health and wellbeing with a summary of which codes were reported in the documents and their quality rating are shown in Table 2. Six were policies, four strategies, two frameworks and one where it met the inclusion criteria, but it was uncertain exactly which document type it was from the title or document content. Eleven of the 14 Health Boards had at least one document with two health boards having both a strategy and policy document. However, three Health boards did not provide any relevant documents. The oldest document dated back to 2013 with four documents published since 2020.

**Table 2:** Summary of documents and quality assessment of codes reported in each document.

	Document Type	Year Published	Goal/Aims	Objectives	Responsibilities	Communication plan	Monitoring Outcomes	Wider Policy Context	Review Date****	Resources
**HB1**	Strategy	2019	Medium	High	High	∼	High	High	High*	X
**HB2**	Framework	2015	High	High	Medium	High*	High	High	Medium*	X
**HB3**	Policy	2013	Low	Medium	High	Low	High	Medium	Medium	X
**HB4**	Policy	2018	High	Medium	High	Low	X	High	High	Medium
**Hb5**	Policy	2020	Medium	X	High	X	X	Medium	High	X
**Hb6(a)**	Strategy	2021	Medium	Medium	Medium	∼	High	Medium	∼	X
**Hb6 (b)**	Policy	2015	Medium	Medium	Medium	High	X	Medium	Medium	X
**Hb7**	Framework	2018	Medium	Medium	Medium	∼	High	High	∼	X
**Hb8**	Policy	2020	Medium	Medium	High	High	X	Medium	High	Medium
**Hb9(a)**	Strategy	2021	High	High	Medium	High*	High	High	∼	High
**Hb9(b)**	Policy	2016	Medium	X	High	X	Medium	Medium	Medium	Low
**Hb10**	Strategy	2015	Low	High	Low	High*	Medium	Medium	Medium*	Low
**Hb11**	Uncertain	2017	Low	Medium	medium	∼	Medium	High	∼	X

x=not provided, ∼=not expected, *=not expected but provided

Of the included documents, all thirteen stated a goal/aim, highlighted responsibilities, and the wider policy context. Eleven stated objectives and nine reported monitoring outcomes. For communication plans, seven documents noted a plan and review dates were provided in nine documents. Five documents made reference to how activities and actions noted in the document would be resourced with eight providing no detail on this.

Good practice and less detailed example extracts for each code are shown in Table 3. Overall the content available for each code varied greatly across documents. *Goals/aims* were generally well articulated across all documents with many providing several clear aims or goals. The level of detail provided varied between documents with some providing quite unspecific goals such as “to improve wellbeing” and others which were more tangible and actionable/implementable goals. Some were clearly presented at the start of the document, whilst others were harder to find through the document. *Objectives* were less clearly articulated in the documents as a whole, with varied levels of detail provided. Detailed objectives provided specific named actions and tasks whilst less clear examples were more high-level processes that should be put in place. Where *responsibilities* were mentioned there tended to be quite a lot of detail provided, typically in relation to specific groups of workers (employees, unions, managers, occupational health, human resources) and gave a clear list of who was expected to do what. Such detail on responsibilities was more evident in those classified as policy documents rather than within the strategies and frameworks.

**Table 3:** Examples of good and poorer content within each code.

Heading	Description of content and good practice example extract	Description of content and less detailed example extract
Goals/aims	Provides a clear list of goals which are quite specific. “*Goal 1 We will provide strategic leadership for staff health and well-being to ensure that this is fully integrated into daily activity* *Goal 2 We will work with staff to provide a continuously improving and healthy working environment* *Goal 3 We will work with staff to improve their physical well-being and enable them to have longer healthier lives* *Goal 4 We will work with staff to improve their mental well-being and reduce workplace stress.”*	Vague goal provided with little detail. “*[Health Board name] will identify and reduce the workplace causes of stress as far as is reasonable practicable and provide a supportive workplace”*
Objectives	Tangible and detailed objectives outlined. “*A toolkit of techniques and approaches will further developed to improve staff mental health and well-being. Specific focus will be given to supporting the long term aspiration of eliminating workplace stress and helping staff to recognise the impact of stress and cope with the effects.”*	Higher level, general objectives noted. “*Reducing sickness absence and enhancing productivity through the management of workplace stress and the promotion of mental health and wellbeing.”*
Responsibilities for action	Clear list of responsibilities by person. Provided in one section of the document and easy to find. For managers one document noted: “*Line managers, senior managers and leaders all have a responsibility for the health, safety and wellbeing of employees while at work and must recognise the impact of good people management on service delivery and organisational performance.”*	Less clear on what each group (employees, mangers etc. should be doing. Notes just notes the role of the main coordinating group. “*We have an established governance group to ensure senior level engagement with this strategy. The Staff Health Strategy Governance Group has lead responsibility for the Strategy.”*
Communication plan	Notes specific actions to communicate plans. “*Circulate Policy via Joint Policy Forum, intranet, Health and Safety/Partnership forums and cascade via management structure, Healthy Working Lives.”*	States communication is important but doesn't provide details on how to achieve it. “*It is important to make communication clear to ensure that employees have realistic expectations of what's possible, practical and affordable.”*
Monitoring outcomes	Provides tangible, detailed outcomes and how they will be measured. “*Progress will be measured through development of a performance framework that will include the following indicators:* *• Number of days lost to sickness absence* *• Number of referrals to OHS* *• iMatters employee engagement index* *• Number of staff with completed PDP* *• Data from annual staff governance statistical report* *• Completion of statutory and mandatory training* *• Stonewall index* *• Completed actions from health and safety plan—dashboard”*	Higher level outcomes noted with little detail of how they will be measured. “*Regular evaluation of staff turnover, sickness absence and stress related incidents identified from the application of other [health board name] and accidents will contribute to the monitoring and reviewing of the policy.”*
Wider context	Mention of several national policies, key reports and legislation in addition to internal health board policies. These may include Staff Governance Standards, Reviews—Boorman, Marmot, Safe and Well at Work: Occupational Health and Safety Strategic Framework for NHSScotland, Health and Safety at Work Act 1974 and letters from the Chief Medical Officer such as CEL01 (2012) and CEL14 (2006).	Mentions other internal health board policies only.
Resources	Provides sources funding as measured outcome and who is responsible for this. “*There is a clear and straight forward process for Work Well Leads to apply for funding”* and notes under the measures section *“number of applications for funding for local initiatives”.*	Mentions resources in terms of funding already again or a statement that resources are needed not detailing how or where they will come from. “*Ensuring that resources are made available to enable the [Health Board name] to be implemented”*

*Communication plans* were mainly vague. Those with the most detail noted that the document would be made available on staff intranet sites and via other channels whilst those with the least detail merely highlighted the need for communication without how this would be achieved. *Monitoring outcomes* included mainly existing data collection methods (as opposed to new ones specifically for the document) and high-level outcomes such as staff turnover, sickness absences, incidences of stress, stress risk assessment, internal staff surveys and feedback. Many also mentioned the NHS Health Scotland IMatter staff experience continuous improvement tool designed with staff in NHS Scotland to help individuals, teams and Health Boards understand and improve the staff experience. Whilst the *wider policy context* was mentioned in all documents the level of detail varied with some making specific reference to key NHS Scotland national documents (such as Chief Medical Officer letters), wider health and safety legislation (e.g., Health and Safety at Work act 1974), reviews and reports [Boorman (15), Marmot (43)], and others referring to just other local Health Board level documents. Of those which made reference to *resources,* the most in-depth documents provided tangible approaches to funding with one having sourcing external funding as an objective with the number of applications made for local initiatives highlighted as a measurable outcome. Those with less detail mentioned that the resource of staff time was needed to progress the actions of the documents with limited additional details. No document mentioned existing monetary resources available to fulfil the actions within the document. No one document reported on all the codes or had high quality ratings across codes making it not possible to provide an overall quality assessment for each document.

When assessing reference to *physical activity*, of the 13 documents, nine mention physical activity (Table 4). Active travel and walking are the most mentioned types of activity; four documents note active travel to work schemes, and five mentioning walking with one noting the importance of taking the stairs. Walking is noted in relation to walk to work schemes, local health walks, step counting campaigns, walking routes, and making use of greenspace to promote walking. The extent to which physical activity is referenced and promoted varies between documents. Eight of the documents reference it with regards to activities they currently deliver with most signposting to physical activity resources that employees can utilise both internally and from external providers. For example, one document mentions local sports and leisure facilities and a potential staff discount fee. Three documents provide more detail on physical activity and note it as a key aim or priority area. These three documents state physical activity as an outcome in relation to active travel/ active travel schemes. All documents, including the three with more physical activity detail, mainly focus on individual level strategies with some, but limited, reference made to environmental changes. None specifically mention physical activity with regards to the workplace culture. Two documents note physical activity change is measured or should be reported as a key outcome.

**Table 4:** Physical activity content for documents where physical activity was referenced.

Health board	Mentions physical activity	Description of content and example extracts *Extracts are provided in italics*	Walking?	Active travel?	PA measured or a key outcome?
HB 1	Yes	Discusses promoting staff to be more active. They make note of a detailed and “*well publicised physical activity campaign”* but doesn't detail this further*.* Notes a specific gardening physical activity programme.	No	No	No (unless in staff survey)
HB 2	Yes	“*Co-ordination of a range of activities designed to enhance and improve well-being at work, for example exercise classes held in some hospital sites, reduced membership costs to local gyms, sports tournaments, resilience and working health matters groups and cycle to work scheme.”*	No	Yes	No (unless in staff survey)
HB 4	Yes	“*All three councils provide sports and leisure facilities in their areas which can be access for a small fee. Further details of these can be accessed from the councils’ websites.”*	No	No	No monitoring of any kind in document
HB 5	Yes	Appendix on counter measures for dealing with stress. It states: “*People can use exercise to stifle the build-up of stress in several ways. Exercise, such as taking a brisk walk shortly after feeling stressed, not only deepens breathing but also helps relieve muscle tension. Movement therapies such as yoga, tai chi and qi gong combine fluid movements with deep breathing and mental focus all of which induce calm.”*	Yes	No	No monitoring of any kind in document
HB 7	Yes	All physical activity content is on active travel. States the Health Board “*aims to create an organisational culture which normalises, and fully supports staff to make travel choices that increase the active component and reduce car use”*. Then states a list of 10 priorities which includes physical activity.	Yes—Active travel	Yes	Not a main objective but states its “*committed to regular review of progress in terms of staff travel choices”*
HB 8	Yes	Provides links to internal service “*including local leisure centres or health walks”* as part of mentally healthy workplace staff support guide infographic. “*Be active”* noted as one of 5 steps to improve mental health. Provide links to local external physical activity provider of health walks in appendix.	Yes	No	No monitoring of any kind in document
HB 9a	Yes	Physical activity mentioned as part of their “*work well pillars”* which notes “*a holistic approach to staying active, healthy and energised!”.* It is noted again in a section on “*Enablers -Environment”* which says: “*Ensure good signage and communication is visible to promote active lifestyles (e.g., encourage staff to take the stairs and stay hydrated)”*.	Yes-Taking stairs example provided	No	No (Unless measured in their wellbeing survey)
HB 10	Yes	Physical activity noted in the introduction as a strategy aim: “*[Health Board name] will ensure that employee's own awareness is increased as to what is important in ensuring their own health and wellbeing needs are met, in particular in relation to the correlation between positive physical, emotional and mental health wellbeing and exercise.”* Mentioned in reference to wider policy. Notes staff are provided with a range of activities such as “*reduced rate exercise classes with local providers by way of corporate membership to local gyms and staff walking initiatives”.*	Yes	Yes	Yes—Increase the uptake of walk and cycle to work schemes
HB 11	Yes	Physical activity noted as a priority area for action. It details the benefits of physical activity and states: “*Workplace wellbeing programmes and facilities to encourage more active travel are a good way to start. Incorporating physical activity is a key component of any programme aiming to reduce the number of obese and overweight employees.”* It then provides evidence of successful interventions from the literature. It notes a pedometer campaign they run and makes specific recommendations for future work including “*Make use of greenspace to promote walking”* and “*Adopt the national Cycle to Work scheme”.*	Yes	Yes	Yes—Active travel survey

## Discussion

The aim of this study was to report on the number of NHS Health Boards in Scotland with workplace health and wellbeing documents for employees; to assess the quality of these documents; and the extent to which these reference lifestyle behaviours, namely physical activity. This study found that despite the importance of a healthy workforce, some Health Boards do not have high-level documents in place to support this. For those that do, there were differences on how many reported the key expected codes set out in this study and the quality of the content within these. Lifestyle factors play a key role in health and wellbeing (44, 45), and when using physical activity as an example, most of the documents included a reference to this. However, these were largely in relation to current activities and signposting employees to resources.

There is the potential to be critical of NHS Health boards for not having documents in place. This is based on the assumption that a specific policy document is needed for action on employee wellbeing to occur in practice. However, it is positive that this study showed that the majority do have these documents in place. One health board that did not have any document retrieved for this study does have resources available for staff wellbeing on their website and it is clear they are taking action in this area without a policy. It is also not possible to suggest from the current results whether having documents with examples of good practice had higher employee health as a result. This debate on policy and action is reflected in a recent commentary discussing the “*policy to practice disconnect”* with regards to physical activity action (46). The authors report on several examples of cities that have created “*comprehensive and robust multisectoral programs and policies”* without formal health policies or leadership in place (47–51). The current study did however find that most documents made reference to higher level, national policies and advice suggesting that higher level polices might act as a drivers for local policy action.

It has been noted in wider literature that without explicit mention of resources (financial, staff time, capacity etc.) how exactly policies and their actions will be enacted is unclear (46). Whilst this study did not assess action as a result of these documents, the lack of mention to resources within the documents is apparent. In the one document with the most detail on how activities would be resourced, reference was made to applying for grants, but still no direct or tangible financial resource were allocated. This information maybe contained in additional internal documents not included in the current review. However, clear reference to resourcing is required to ensure actions can be taken forward and is a key recommendation for future policy and healthcare documentation development work.

This study also found that there were large variations in content between the documents. This is with reference to whether the expected codes were mentioned, the quality of the content (strength and comprehensiveness) and the ease of finding the relevant content. Out of the eight key codes expected to be reported in these documents, only three were reported in every document. The difference between good practice and less detailed examples demonstrate the disparities between these documents. Again, whilst inferences cannot be made with regards to what difference this might make to potential implementation and improvements in employee health as this was not explored, these codes were developed from existing tools aimed to develop and/or assess policies and therefore likely to be important for document quality and potential implementation and effect. Producing standard guidance including good practice examples for health boards could ensure the documents have a greater chance of being impactful.

Given the benefits of a healthy lifestyle for employees (52), having 70% of the documents mentioned physical activity is encouraging. However, due to the importance of physical activity in relation to health, it might be expected for all Health Boards to include it in their health and wellbeing documents. Those that did mention physical activity, did so in relation to existing activities as opposed to future plans, and signposting to existing, external resources to help people become more active, more than proactive strategies implemented by the workplace themselves. The content was also very individually focused (as opposed to addressing the wider physical activity environment) such as including walking groups and encouraging people to cycle into work. This lack of depth is not surprising given the evidence-base is largely built on these types of individual interventions and the ease with which they can be deliver in comparison to more complex interventions (20). These documents could instead go beyond individual level interventions and focus on the context around individuals (physical, social, cultural, policy environment) and how these interconnect. Reference to tools such as the Action Scales Model (53) which help policymakers to plan how to intervene more efficiently in a system could be useful for future practice. In addition, many of these physical activity suggestions focus on active leisure time or active travel which may be especially challenging to promote in a workforce where many people are on their feet all day. There was no mention of the role of occupational activity in these documents or even that for some groups, more rest may be needed over more movement. Future policies need to take into account the complexity of the health care workforce in this regard and to ensure their involvement and co-design in both policy and intervention development.

Measurement of lifestyle behaviours such as physical activity could be used as a means to gain further support for these policies and to monitor their effectiveness. Despite there being 13 documents, only 11 noted outcomes, and of these most were high level, routinely collected outcomes such as sick leave. Whilst measures such as sick leave are useful as systems are already in place to capture the data, they are unlikely to change in a short period of time and influenced by multiple complex factors; therefore, difficult to attribute change as a result of organizational policy. More responsive measures such as physical activity or other lifestyle factors could be included as outcomes in order to assess potential change from employee health and wellbeing documents.

This study should be used as starting point with regards to reviewing health and wellbeing policies for health care employees. Whilst a mainly descriptive analysis was presented in this study, it is possible to see how improvements in these documents within the codes assessed could be more likely to lead to action on employee health. Whether policy leads to action and whether higher quality policies lead to action is an area of future research with regards to employee health and wellbeing in the health care workforce. In addition, the methods used in the study provide an initial framework for the analysis of health and wellbeing policy documents for health care employees which could be developed further to be replicable both nationally and globally. There was limited guidance on what to include in analysis of these high-level documents and whilst the methods used here built on existing research and good practice, the assumptions and limitations of this framework should be critiqued in future research and a clear focus on quality assessment further developed. This study also focused on one specific lifestyle behaviour, physical activity and wider lifestyle behaviours such as smoking, and diet were not assessed. Future studies should conduct similar analysis and assess additional lifestyle behaviours namely diet. Whether having an organizational policy in place leads to better improvements in employee health is still unclear, and whilst difficult to assess, future research could begin to explore the effects of such policies on health and wellbeing.

## Conclusion

Despite the importance of a healthy health care workforce there are not always documents available to support these efforts. When documents were available, they varied greatly with regards to what was reported and the quality of the included content. With reference to lifestyle behaviours, using physical activity as an example, most documents mention physical activity but with limited detail and mainly in relation to current activities rather than in the context of a future healthy workforce. More work is needed to ensure high level, national documents are available to support health care employee's health and wellbeing. Ensuring the quality of these documents by creating standard templates and making reference to lifestyle behaviours such as physical activity could be important for creating a healthy health care workforce.

## Data Availability

The original contributions presented in the study are included in the article/Supplementary Material, further inquiries can be directed to the corresponding author.
